# Socially Assistive Robot *Hyodol* for Depressive Symptoms of Community-Dwelling Older Adults in Medically Underserved Areas: A Preliminary Study

**DOI:** 10.3390/jcm15010217

**Published:** 2025-12-27

**Authors:** Han Wool Jung, Yujin Kim, Hyojung Kim, Min-kyeong Kim, Hyejung Lee, Jin Young Park, Woo Jung Kim, Jaesub Park

**Affiliations:** 1 Department of Psychiatry, Yongin Severance Hospital, Yonsei University College of Medicine, Yongin 16995, Republic of Korea; 2 Department of Integrative Medicine, Yonsei University College of Medicine, Seoul 06229, Republic of Korea; 3 Graduate School of Public Administration, Seoul National University, Seoul 08826, Republic of Korea; 4 Institute of Behavioral Science in Medicine, Yonsei University College of Medicine, Seoul 03722, Republic of Korea; 5 Center for Digital Health, Yongin Severance Hospital, Yonsei University College of Medicine, Yongin 16995, Republic of Korea

**Keywords:** socially assistive robots, social robots, humanoid robots, person-centered approach, medically underserved areas, user acceptance, mental health in older adults

## Abstract

**Background/Objectives**: Socially assistive robots effectively support elderly care when they incorporate personalization, person-centered principles, rich interactions, and careful role setting with psychosocial alignment. *Hyodol*, a socially assistive robot designed for elderly people, embodies a grandchild’s persona, emulating the grandparent–grandchild relationship. Based on the behavioral activation principles and a human-centered approach, this robot continuously supports users’ emotional well-being, health management, and daily routines. **Methods**: The current study evaluated *Hyodol’s* impact on depressive symptoms and other quality of life factors among older adults living in medically underserved areas. A total of 278 participants were assessed for depressive symptoms, loneliness, medication adherence, and user acceptance. **Results**: After six months of use, participants showed significant reductions in overall depressive symptoms, with a 45% decrease in the proportion of individuals at high risk of depression. Significant improvements were also observed in loneliness and medication adherence. Participants reported high levels of user acceptance and satisfaction, exceeding 70% of the total score. Participants who engaged more frequently in free chat with *Hyodol* showed greater improvements in depressive symptoms. **Conclusions**: These results highlight *Hyodol’s* potential as a promising tool for enhancing mental healthcare and overall well-being in this population. This at-home mental-healthcare framework can complement primary care and, if its effects are confirmed in controlled trials, could contribute to reducing healthcare burden and preventing the onset and escalation of depressive symptoms.

## 1. Introduction

Digital health technologies are increasingly being adopted to address mental health challenges of community-dwelling older adults, especially in medically underserved regions. Challenges among community-dwelling older adults include depression arising from social isolation and loneliness, sleep disturbances, cognitive decline, and increased suicide risk [1,2,3,4,5,6,7,8]. Older adults residing in medically underserved areas, defined as regions with restricted availability of primary healthcare [9,10], may be especially vulnerable, as they often experience limited access to mental healthcare services [11,12]. Digital and mobile remote health solutions can effectively enhance mental health outcomes among individuals in medically underserved areas [13]. Studies suggest that digital technologies have significant potential for managing depression, anxiety, insomnia, and psychotic symptoms, demonstrating high acceptability and completion rates. However, constraints remain, such as limited connectivity, privacy concerns, and a need for more rigorous clinical trials [13,14,15].

Among these cutting-edge solutions, socially assistive robots (SARs) have recently been deployed in home care settings to address loneliness and depression in community-dwelling older adults [16,17,18]. SARs support older adults through social interactions and effectively promote positive emotions, encourage meaningful activities, improve social functioning and quality of life, and reduce symptoms such as depression and loneliness [19]. Despite promising initial results, however, acceptance of SARs varies considerably among older adults, highlighting the need for personalized robot design strategies to foster individual engagement [20]. For improved engagement and effectiveness in elderly care, SARs should integrate people-centric principles with rich, multimodal interactions. Moreover, tailoring the robot’s persona to match user motivations, such as addressing loneliness or the desire for close companionship, is crucial for effective care, and should incorporate thoughtful design cues developed through a user-centered approach [19,21].

*Hyodol* is a SAR specifically designed to address these challenges. Designed for elderly care, it embodies the persona of a loving grandchild. With its doll-like appearance resembling a young boy or girl, this robot provides daily support through continuous, friendly conversation. These ongoing interactions help manage various aspects of a user’s well-being, including life and health monitoring, emotional support, and safety management. Its warm, human-like presence aims to foster a sense of companionship, thereby enhancing mental health and quality of life for older individuals.

**Figure 1:**
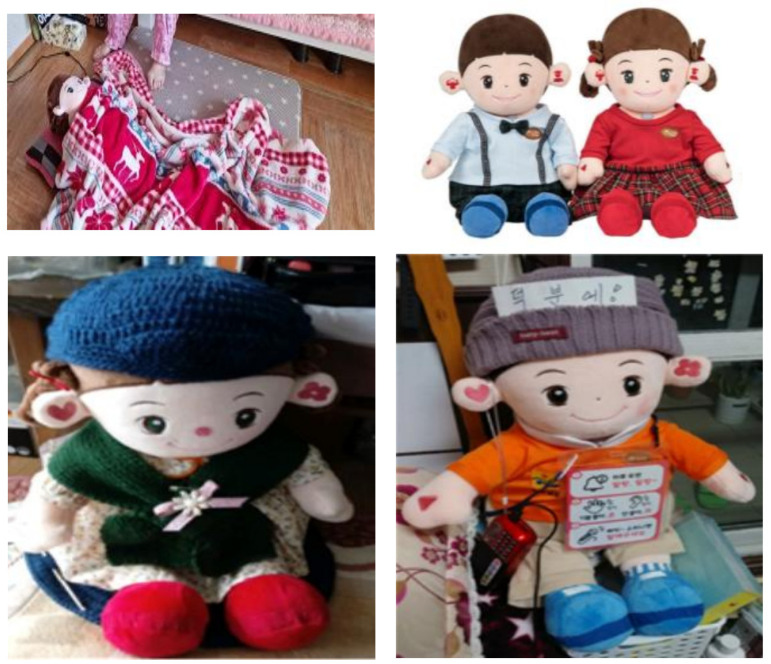
Appearance of *Hyodol* [27] (the robot used in the present study; (**top right**)) and expressions of user intimacy. Users who feel a strong sense of closeness toward *Hyodol* often demonstrate this by covering the robot with blankets (**top left**), knitting them clothing and hats (**bottom left**), or attaching notes such as “Thanks to you!” (**bottom right**).

*Hyodol* is designed to foster intimacy with users by serving as a grandchild-like companion, in both appearance and interaction. Many users form strong attachments, treating it as they would a real grandchild—showing affection, making clothes, or tucking it in at night [22] (Figure 1). Building on this rapport, *Hyodol* supports users in maintaining healthy routines, such as timely medication, regular meals, physical exercise, and cognitive activities. This strategy is rooted in behavioral activation, which emphasizes monitoring and reinforcing healthy behaviors, which can effectively manage psychological symptoms like depression [23]. Additionally, *Hyodol* employs a personalized, human-centered approach, using artificial intelligence and Internet of Things (IoT) technologies to create adaptive, user-friendly care tailored to individual needs.

This distinctive feature of *Hyodol* may offer merits over other SARs. Previous SARs have raised concerns regarding sustained engagement and acceptance [24,25]. Moreover, many SARs have primarily focused on companionship, with limited integration of daily routines, behavior change strategies, or the promotion of healthy behaviors, which may contribute to uncertain long-term effectiveness [25,26]. In contrast, *Hyodol* was designed to foster perceived intimacy through a distinct persona and role, while also supporting personalization and human-centered care via daily health-support functions informed by user engagement. These features may help address engagement-related limitations of prior SAR-based interventions and support sustained usability and potential health benefits, particularly in home deployment among community-dwelling older adults with limited access to traditional mental healthcare services.

**Figure 2:**
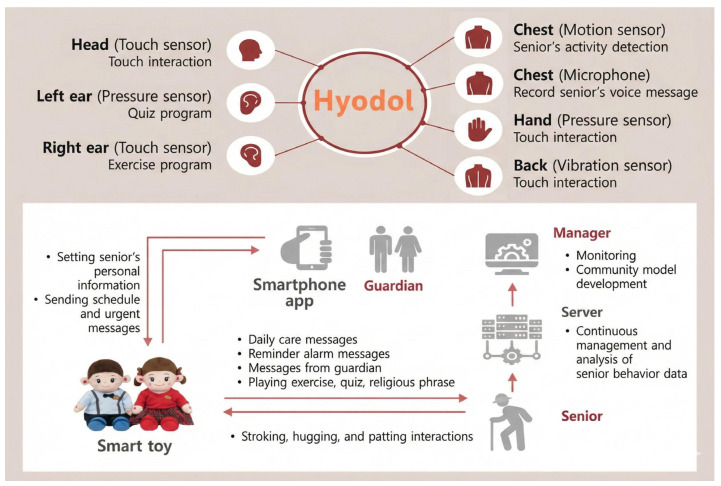
Technical features of *Hyodol*. Sourced from Yoo et al. [27] (JMIR Publications) under the Creative Commons Attribution 4.0 (CC BY 4.0) license; redrawn by the authors using Gemini 3.

*Hyodol* incorporates a range of interactive features. Sensors on its head, hands, ears, and body respond to touch and movement, while microphones and motion sensors monitor speech and behavior. The robot also conducts daily check-ins on users’ mood, pain, meals, medication, and sleep patterns, thereby supporting behavioral activation. Its content library spans singing, exercise, quizzes, and religious materials, addressing various user interests. *Hyodol* also features a free interaction mode, enabling spontaneous conversations and responses to both verbal and physical cues, which fosters long-term engagement. Finally, it provides real-time health monitoring, which can promptly detect users’ current status. All monitoring data are shared with their guardians and local welfare/community centers through a smartphone or PC app, ensuring that timely support and interventions are available when necessary. The detailed technical features of *Hyodol* are available in Figure 2.

Studies on *Hyodol* have shown promising results. Qualitative studies indicate that users appreciated not only its coaching role but also the emotional support and companionship it provides, which brings them pleasure and reduces loneliness. Users even reported that they regarded *Hyodol* as a friend or even a family member [28,29]. Additionally, a recent empirical study found that interventions using *Hyodol* significantly reduced depressive symptoms among older adults, although the effect was less pronounced during the COVID-19 pandemic compared to the pre-pandemic period. While the reason for this discrepancy is unclear, the authors conjectured that the additional psychological burden during the pandemic may have influenced the results [30]. Despite these encouraging findings, these results call for further research to more robustly confirm *Hyodol*’s efficacy, ideally with a larger sample size.

The current study aims to assess the impact of *Hyodol* in medically underserved areas, particularly focusing on rural regions or communities with healthcare disparities. We anticipate that robots like *Hyodol* can provide continuous, proactive care through physical and psychological proximity and could significantly complement primary care services within these underserved communities. *Hyodol’s* features, such as remote patient monitoring, real-time decision support, telemedicine, and mobile sensing, have the potential to enhance healthcare accessibility in these underserved settings [31,32,33]. Building on previous studies that focused on socially isolated older adults and cohorts during the pandemic [24,26], this study examines the extent to which *Hyodol* improves depressive symptoms and psychological well-being among community-dwelling older adults in medically underserved areas.

## 2. Materials and Methods

### 2.1. Participants

Participants were those who provided consent to receive non-face-to-face remote mental healthcare services, selected from among community-dwelling older adults aged 65 years or older living in rural and medically underserved areas, who had been using *Hyodol* in their homes along with visiting home care services for the elderly. Participants were selected retrospectively after *Hyodol* had already been deployed in their homes, and only those who intended to receive mental healthcare services were included. All participants provided written informed consent to use the survey data for analysis as part of the consent for receiving remote mental healthcare services. Among the participants who agreed to participate, those without valid survey data for both baseline and follow-up were excluded.

### 2.2. Measures

#### 2.2.1. The Geriatric Depression Scale-Short Form (GDS-S)

The GDS-S [34] is a self-reported instrument designed to assess depression in older adults. While the original scale contains 30 items, the short form consists of 15 items. Each item is answered in a binary format (0 or 1). For scoring, 10 items are coded as 1 when answered “yes,” while the remaining five items are coded as 1 when answered “no.” The GDS-S score ranges from 0 to 15. Although Greenberg et al. (2007) suggested that scores of 10 or higher are almost always indicative of depression [35], we used this cutoff to indicate a high risk of depression rather than to define a definitive diagnosis, because this tool is intended as a screening instrument rather than a formal diagnostic tool. Participants with scores at or above this threshold were classified as a high-risk group for depression in this study.

#### 2.2.2. Patient Health Questionnaire-9 (PHQ-9)

The PHQ-9 [36] is a nine-item scale for the screening of depression, with each item rated on a 4-point Likert scale from 0 to 3. The total score, ranging from 0 to 27, is calculated by summing all item scores. Higher scores indicate more severe depressive symptoms. PHQ-9 scores below 4 are almost always indicative of the absence of depression; scores of 5–9 represent either no depression or subthreshold/other types of depressive symptoms; scores of 10–14 represent a heterogeneous spectrum of patients; and scores of 15 or higher usually indicate major depression [36].

#### 2.2.3. UCLA Loneliness Scale

The 20-item UCLA Loneliness Scale [37] assesses participants’ social isolation and feelings of loneliness. Each item is rated on a 4-point scale, ranging from 1 (never) to 4 (always), resulting in a total score ranging from 20 to 80. Higher scores indicate greater levels of loneliness.

#### 2.2.4. Medication Adherence

We assessed medication adherence using a 5-point Likert scale (ranging from 0 to 4), measuring whether participants forgot to take their medication, missed doses, took medication at doses different from those prescribed, or did not take their medication for various reasons. Item scores were summed to yield a total score ranging from 0 to 20, with higher scores indicating greater adherence.

#### 2.2.5. User Acceptance

This 24-item part measure assessed participants’ overall satisfaction, positive experiences, negative experiences (reverse coded), frequency of use, and perceived ease of use of the robot, using a 5-point scale ranging from 0 to 4. The scale was adapted from the Almere model [38] for measuring user acceptance of socially assistive technology among older adults. This model assesses perceived usefulness, perceived ease of use, satisfaction/attitude toward using, intention to use, feelings of trust/sociability/warmth, enjoyment, and anxiety/negative attitudes, based on the technology acceptance model [39] and the Unified Theory of Acceptance and Use of Technology [40]. The item scores were averaged.

#### 2.2.6. Usage Logs

Usage data from *Hyodol* were recorded throughout the user engagement period. It includes user responses to *Hyodol*’s daily health check-in questions (capturing user sentiments), the daily frequency of users initiating free chat with *Hyodol*, the frequency of physical interactions with *Hyodol* (e.g., stroking, tapping, or knocking the robot), and the frequency of content usage from *Hyodol*’s library (e.g., memory, brain activity, music, exercise, religious content). These records are considered indicators of active *Hyodol* use and engagement, providing insights into individual usage patterns.

### 2.3. Procedure

Initial surveys were conducted between 19 December 2023 and 11 March 2024, when participants began using *Hyodol* and receiving home care services. The participants were given detailed instructions on how to use *Hyodol* at the time of initial deployment by caregivers affiliated with local welfare centers. Following an approximately six-month intervention period, during which usage logs were recorded, follow-up surveys were held from 1 July to 13 November 2024. The intervention period varied across participants, as the data were collected in real-world settings across multiple deployment sites. The participants also received remote video interview sessions for the assessment and counseling of their depressive symptoms. These sessions included a structured clinical interview conducted by psychological researchers, followed by a second interview for selected participants conducted by a board-certified psychiatrist specializing in geriatric psychiatry. Some participants were diagnosed with significant depressive symptoms through these sessions.

**Figure 3:**
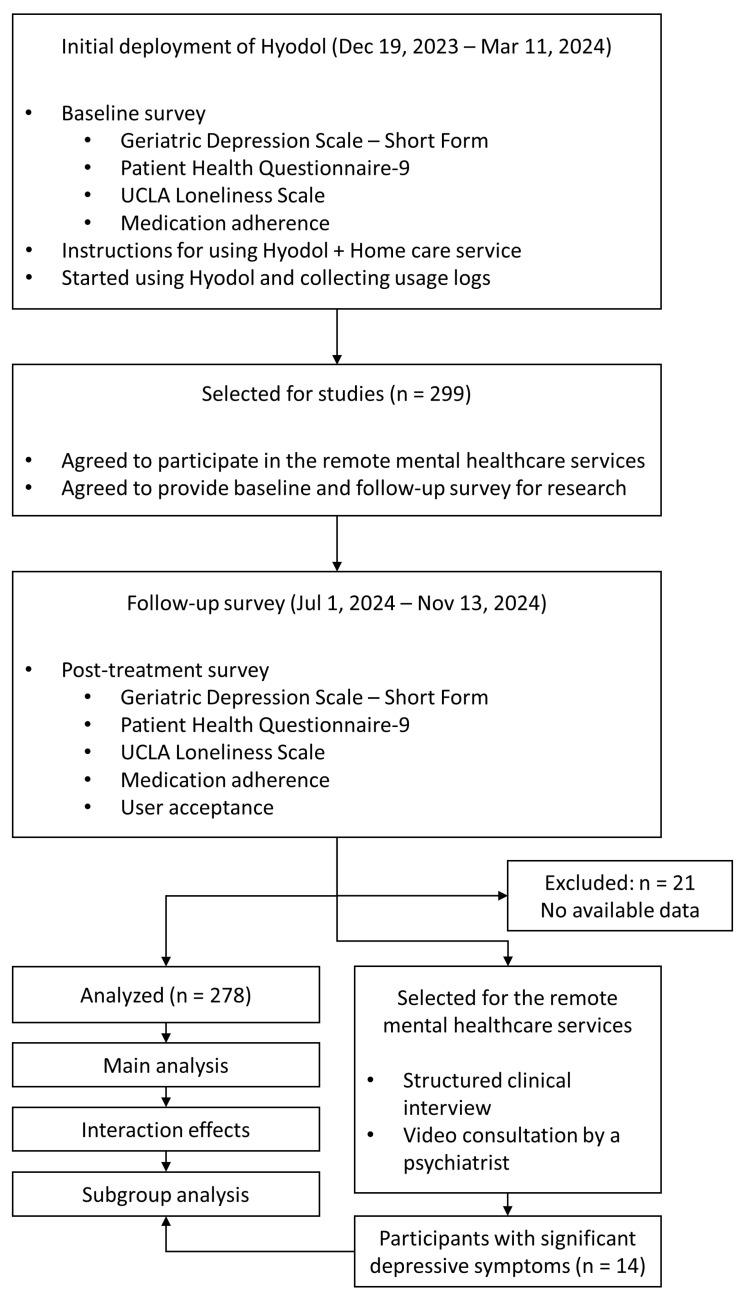
Study flow diagram.

The efficacy of the robot and the robot-assisted home care services was evaluated by measuring changes in depressive symptoms, loneliness, and medication adherence between the initial and follow-up surveys. Changes in the proportion of participants classified as the high-risk group for depression (based on GDS-S) between the initial and follow-up stages were also assessed. User acceptance was only assessed during the follow-up period to evaluate participants’ experiences and satisfaction levels. The interaction effects of usage logs were also tested to identify the relationship between intervention effects and usage patterns. The overall procedure of the study is illustrated in Figure 3.

### 2.4. Statistical Analysis

Pre-post differences across all participants were analyzed using a single-arm design without a control group. Paired *t*-tests were used to assess changes in raw scores, while McNemar’s test with Yates’ continuity correction was applied for categorical analysis. Parametric statistics were used under the assumption that the sample size was sufficiently large for the central limit theorem to apply. Data were excluded if neither initial nor follow-up responses were available. If only one of the initial or follow-up responses was available, the missing value was imputed using the available counterpart (i.e., the initial value was carried forward to the missing follow-up value, and vice versa). This way of imputation is considered equivalent to the methods assuming no change over time for those cases (e.g., last observation carried forward [LOCF] or next observation carried backward [NOCB]), which is a conservative approach that does not introduce artificial improvement and, if anything, tends to attenuate pre-post differences and bias the results toward the null hypothesis rather than inflating statistical significance.

In addition to the main analysis, interaction effects between *Hyodol* usage variables and the pre-post change in the primary depressive symptom measure (GDS-S) were analyzed using repeated-measures ANCOVA. Subgroup analyses were performed for each measure among participants clinically diagnosed with significant depressive symptoms during remote mental healthcare sessions. Throughout the analysis, within-group (pre-post) changes in the measures with standard deviations (*SD*s) were reported with the effect sizes with their 95% confidence intervals (CIs) additionally reported.

## 3. Results

### 3.1. Participants

A total of 278 participants were included in the study, out of 299 older adults who provided valid consent for remote mental healthcare services. Participants’ mean age was 80.7 ± 5.6 years (range: 67–93). The majority were female (88.5%), and most had either no formal education (50.4%) or had only graduated from elementary school (38%). A high proportion of participants were bereaved of their spouse (80.1%) and were living alone (93.1%), indicating that this group represents a vulnerable population requiring at-home primary care.

### 3.2. Depressive Symptom Levels

**Figure 4:**
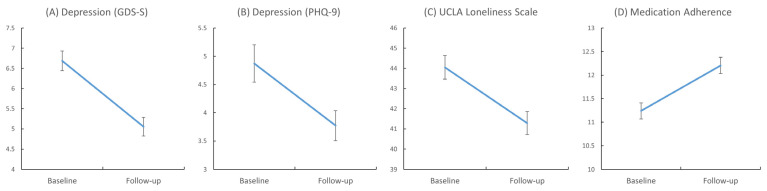
Within-group changes from baseline to follow-up in GDS-S (**A**), PHQ-9 (**B**), UCLA Loneliness Scale (**C**), and medication adherence (**D**). Error bars represent standard errors. GDS-S, Geriatric Depression Scale-Short Form; PHQ-9, Patient Health Questionnaire-9; UCLA, University of California Los Angeles.

At follow-up, the mean GDS-S score decreased from 6.69 to 5.05 (*p* < 0.001; Hedges’ *g* = 0.41, 95% CI [0.29, 0.54]). The mean PHQ-9 score decreased from 4.87 to 3.77 (*p* < 0.001; Hedges’ *g* = 0.22, 95% CI [0.10, 0.35]). The number of participants categorized into the GDS-S high-risk group decreased by 45%, from 92 at baseline to 51 at follow-up (*p* < 0.001; odds ratio = 3.16, 95% CI [1.89, 5.29]) (see Table 1 and Figure 4).

### 3.3. Loneliness and Medication Adherence

Participants’ UCLA Loneliness Scale scores decreased from 44.0 to 41.3 (*p* < 0.001; Hedges’ *g* = 0.29, 95% CI [0.17, 0.40]). The average medication adherence score increased from 11.2 to 12.2 (*p* < 0.001; Hedges’ *g* = 0.34, 95% CI [0.19, 0.48]).

### 3.4. User Acceptance

Participants reported a mean user acceptance score of 2.89 out of 4 (*SD* = 0.67). Focusing specifically on 12 items related to satisfaction, the mean user satisfaction score was 3.16 out of 4 (*SD* = 0.78), indicating a high level of satisfaction with *Hyodol*.

### 3.5. Interactions with Usage Logs

Among the *Hyodol* usage log variables, the daily frequency of initiating free chat with *Hyodol* showed a significant interaction with time on GDS-S scores, as assessed by repeated-measures ANCOVA, *F*(1, 168) = 4.79, *p* = 0.030, partial η^2^ = 0.028 (95% CI [0.000, 0.093]). Participants with a free chat frequency of less than −1 *SD* showed a mean ± *SD* pre-post change in depressive symptoms (ΔGDS-S) of 1.03 ± 4.40 (*n* = 22), those within −1 to +1 *SD* had a change of 2.24 ± 3.70 (*n* = 127), and those greater than +1 *SD* had a change of 4.74 ± 4.56 (*n* = 21), indicating greater symptom reductions among participants who engaged more frequently in free chats. No other usage log variables demonstrated significant interaction effects with the change in GDS-S.

### 3.6. Subgroup Analysis

Subgroup analysis was conducted for 14 participants clinically diagnosed with significant depressive symptoms during remote mental healthcare sessions. Unlike the main analysis, no significant changes in depressive symptoms were observed for GDS-S (9.32 ± 3.66 to 8.14 ± 3.44, *p* = 0.374; Hedges’ *g* = 0.31, 95% CI [−0.37, 0.98]) and PHQ-9 (5.12 ± 6.07 to 6.86 ± 5.35, *p* = 0.335; Hedges’ *g* = −0.29, 95% CI [−0.85, 0.29]). The number of GDS-S high-risk group participants changed from 9 (64%) to 6 (43%), *p* = 450; odds ratio = 2.5, 95% CI [0.49, 12.89]. No significant changes were observed for UCLA Loneliness Scale (49.14 ± 7.65 to 48.36 ± 6.63, *p* = 0.599; Hedges’ *g* = 0.10, 95% CI [−0.28, 0.48]) or medication adherence (11.07 ± 3.12 to 12.21 ± 2.72, *p* = 0.218; Hedges’ *g* = 0.37, 95% CI [−0.21, 0.94]). This group reported a user acceptance score of 2.61 (*SD* = 0.65) and a user satisfaction score of 2.85 (*SD* = 0.70).

## 4. Discussion

The current study suggests that the innovative SAR, *Hyodol*, may improve depressive symptoms and overall mental health in older adults in medically underserved areas. The robot’s persona, designed to mimic a beloved grandchild, fosters a sense of intimacy with users, enhancing engagement in behavioral activation and other supportive programs. Over approximately six months of robotic intervention, participants experienced significant reductions in depressive symptoms and loneliness, alongside improved adherence to prescribed medications. Notably, this study showed a significant reduction in the proportion of participants classified as high-risk for depression (GDS-S scores of 10 or higher), raising the possibility that *Hyodol* could help reduce the burden on intensive mental healthcare and serve as an effective primary care adjunct for older adults, particularly in medically underserved areas. The effect sizes are interpreted as small to moderate but clinically meaningful improvements in depressive symptoms, loneliness, and medication adherence among community-dwelling older adults. However, these findings should be interpreted as preliminary and hypothesis-generating rather than definitive.

Beyond the overall pre-post improvements, additional analyses revealed significant interaction effects between the time effect and the usage data indicating active user engagement. Specifically, the frequency of free chat initiation with *Hyodol* was significantly associated with changes in GDS-S scores over time, such that participants who engaged in more frequent spontaneous interactions exhibited greater reductions in depressive symptoms. This finding suggests that the effectiveness of SARs may depend not only on exposure or duration of use, but also on the extent to which users actively and voluntarily engage with the device. In this regard, *Hyodol*’s ability to elicit spontaneous, emotionally motivated interaction may be a particularly important mechanism for facilitating behavioral activation and subsequent improvements in mood among older adults. However, these interaction effects should be interpreted as exploratory and contributory rather than confirmatory or definitive, underscoring the multifactorial nature of late-life depressive symptoms.

This study represents one of the largest investigations to date examining the effects of SARs on older adults’ mental health, particularly depressive symptoms. This study offers valuable insights into the potential of remote and automated mental healthcare using SARs, highlighting a promising avenue for addressing gaps in mental health services for older adults, although the current findings require further confirmations. SARs like *Hyodol* could enhance mental healthcare accessibility, particularly in remote or underserved regions, effectively supplementing traditional healthcare approaches. Additionally, the engaging and personalized nature of SAR interactions may help reduce the stigma associated with seeking mental health treatment, encouraging older adults to proactively manage their mental health conditions. If supported by further controlled trials, SARs could contribute to improvements in the accessibility, responsiveness, and effectiveness of primary healthcare services, helping to address critical service gaps prevalent in rural or underserved communities [10,11].

However, caution is warranted when generalizing these findings to primary care settings for older adults presenting with depressive symptoms. Although digital interventions for depression generally demonstrate effectiveness across various symptom severities and clinical stages [41], treatment outcomes vary significantly depending on depression severity and the presence or absence of therapeutic guidance. Specifically, guided digital interventions involving human therapists have been more effective in individuals with severe or chronic depression, while self-guided or automated interventions are more suitable and effective for individuals experiencing mild or early-stage depression [42]. In the current study, subgroup analysis involving only participants clinically diagnosed with significant depressive symptoms during remote mental healthcare sessions revealed no significant changes in depressive symptoms and loneliness. While a modest, albeit insignificant, reduction in the proportion categorized as high-risk by the GDS-S was observed, these results highlight the presence of older adults with clinically diagnosed depression who are less responsive to *Hyodol* intervention alone.

Therefore, standalone *Hyodol*-based interventions might be more effectively targeted toward older adults with subclinical, mild, or early-stage depressive symptoms. For individuals with moderate-to-severe depression, integrating supplementary treatments, such as pharmacotherapy or additional care strategies, may be necessary for a more comprehensive and personalized therapeutic approach [43]. Moreover, even when limiting the target group to community-dwelling older adults at risk of depression or in the early stages of depressive symptoms, additional limitations arise in confirming *Hyodol’s* effectiveness. For instance, the current study is a preliminary observational study without a stringent clinical protocol. The absence of a randomized controlled trial (RCT) design, the lack of a control group, and the potential confounding effects such as seasonal effects and home-care services provided concurrently with *Hyodol* also limit the strength of causal inferences that can be drawn from this study. The intervention period was not standardized and varied across participants, and participants were selected only from those who consented to receive mental healthcare, potentially introducing selection bias. Finally, the generalizability of *Hyodol*’s efficacy may be influenced by cultural factors; variations in the importance and expression of family values and nurturing could impact how older adults respond to a SAR designed with a grandchild’s persona.

Despite challenges in obtaining large-scale, representative data from older adults in remote areas or those unfamiliar with computerized surveys, future research should aim to replicate and generalize these findings across diverse populations and different SARs. This should ideally be conducted through randomized controlled trials employing valid comparators and pre-registered protocols. Ultimately, evidence from rigorous studies emphasizes SARs’ promising role in expanding access to mental health support and improving the quality of life for older adults in medically underserved areas.

## Figures and Tables

**Table 1:** Within-group score changes.

Measures	Baseline (M ± SD)	Follow-Up (M ± SD)
GDS-S	6.69 ± 4.08	4.08 ± 3.83
GDS-S high risk (*n*)	92 (33%)	51 (18%)
PHQ-9	4.87 ± 5.53	5.53 ± 4.43
UCLA Loneliness	44.05 ± 9.72	9.72 ± 9.49
Medication adherence	11.24 ± 2.85	2.85 ± 2.90
User acceptance	–	2.89 ± 0.67
User satisfaction	–	3.16 ± 0.78

Note: All within-group effects were statistically significant (*p*s < 0.001). GDS-S, Geriatric Depression Scale-Short Form; PHQ-9, Patient Health Questionnaire-9; UCLA, University of California Los Angeles; M, mean; SD, standard deviation.

## Data Availability

The datasets generated and analyzed during the current study are not publicly available due to privacy and ethical restrictions but are available from the corresponding author upon reasonable request.
